# The Relationship Between Vitamin D and Asthma Exacerbation

**DOI:** 10.7759/cureus.17279

**Published:** 2021-08-18

**Authors:** Opemipo D Ogeyingbo, Rowan Ahmed, Mallika Gyawali, Nanditha Venkatesan, Renu Bhandari, Rinky A Botleroo, Roaa Kareem, Abeer O Elshaikh

**Affiliations:** 1 Research, California Institute of Behavioral Neurosciences & Psychology (CIBNP), Fairfield, USA; 2 Public Health, Walden University, Minneapolis, USA; 3 Internal Medicine, Saint James School of Medicine, Park Ridge, USA; 4 Internal Medicine, California Institute of Behavioral Neurosciences & Psychology (CIBNP), Fairfield, USA; 5 Internal Medicine, All India Institute of Medical Sciences, Raipur, IND; 6 Internal Medicine/Family Medicine, California Institute of Behavioral Neurosciences & Psychology (CIBNP), Fairfield, USA; 7 Internal Medicine, Manipal College of Medical Sciences, Pokhara, NPL; 8 Medicine, California Institute of Behavioral Neurosciences & Psychology (CIBNP), Fairfield, USA

**Keywords:** asthma, asthma exacerbation, wheezing, vitamin d, bronchoconstriction

## Abstract

Asthma is a chronic airway inflammatory condition that affects millions of people worldwide. It presents with reversible bronchoconstriction that makes it difficult for patients to breathe. Asthma flare-ups have several triggers, but the symptoms are similar, including wheezing, coughing, shortness of breath, and chest tightness. Severe asthma exacerbation is described as symptomatic asthma that is unresponsive to inhaled asthma medications and is only responsive to steroids in oral or intravenous forms. Asthma-related deaths occur during episodes of asthma exacerbation.

Vitamin D is a steroid-derived vitamin produced by the body and found in some foods. Administration of doses of vitamin D can also help maintain an adequate level of the vitamin. Vitamin D plays a vital role in regulating the level of calcium in the body and bone remodeling processes. It also has an immunomodulatory effect on innate and adaptive immunity within the body and that partially explains its links to inflammation-induced epithelial changes seen in asthma.

We conducted this literature review by selecting articles from PubMed and Cumulated Index to Nursing and Allied Health Literature (CINAHL) Plus databases to investigate the relationship between vitamin D level and asthma exacerbation. From the studies, we found that asthmatic patients have low vitamin D levels during an asthma exacerbation. However, supplementing vitamin D may not reduce the rates of asthma exacerbation except in adult asthmatic patients with low levels of vitamin D.

## Introduction and background

**Figure 1:**
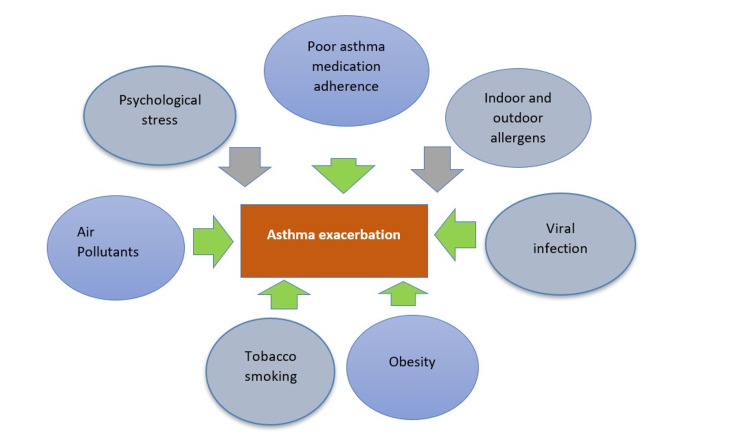
Triggers of asthma exacerbation

Asthma is a chronic airway inflammatory condition that has several triggers, including viral infections, obesity, psychosocial stress, air pollutants, stress, tobacco smoke, indoor and outdoor allergy sources, as shown in Figure 1, as well as the poor use of asthma maintenance medications; and it affects people of all ages [1,2]. Severe asthma that is unresponsive to daily maintenance medications is called asthma exacerbation. It presents as severe shortness of breath, chest tightness, coughing, and wheezing that is only responsive to systemic corticosteroids such as oral prednisone or intravenous corticosteroids [3].

Over 300 million people worldwide have asthma, and the number of deaths per year related to asthma is around 400,000 [4]. Most asthma-related deaths occur during asthma exacerbations when escape medications fail to control the symptoms of airway constriction [4]. The lethality associated with asthma exacerbations highlights the importance of studying asthma and conditions limiting its flare-up [5]. Chronic inflammation from asthma modulated by immune cells, T-helper (Th) cells, leads to activation of mast cell and immunoglobulin E (IgE) and contribute to further inflammation of the airways, creating wheezing, chest tightness, and cough symptoms [2].

Cholecalciferol (vitamin D) is a steroid-derived vitamin produced in the body, mainly in the skin, in the form of vitamin D3, a precursor derived from sunlight exposure of 7-dehydrocholesterol; other important vitamin D sources are fish rich in fat, fish liver oils, white cheese, egg yolks, and beef liver [6]. Vitamin D, known for its calcium absorption and bone modeling function, also has an immunomodulatory effect on innate immunity and adaptive immunity within the body, which explains its links to inflammation-induced epithelial changes in asthma [1,7]. The research found that patients with low vitamin D were more at risk for non-influenza upper respiratory conditions. At the same time, low vitamin D also increases the risk of progressive lung diseases, and supplementing vitamin D is beneficial in both conditions [6,8-10]. Several studies have also associated low vitamin D with asthma flare-ups, poor lung functions, and ineffectiveness of asthma medications [2,7].

The primary goal of this literature review is to examine the connection between asthma exacerbation and vitamin D levels. We gathered fifteen scholarly articles, with a total of 9,527 participants, from PubMed and Cumulated Index to Nursing and Allied Health Literature (CINAHL) Plus databases based on relevance to this topic. We included full-text peer-reviewed scholarly articles from May 2015 to May 2021 with children and adults as participants. We included randomized controlled trials, systematic reviews, metanalysis, cohort studies, and traditional reviews.

## Review

Relationship between vitamin D levels and asthma exacerbation

Turkeli et al. conducted a cross-sectional study where 115 children with asthma and 115 controls were selected from the total number of patients who went to pediatric immunology and allergy outpatient clinic over three months; patients with low levels of vitamin D (vitamin D level less than 20ng/mL) were more likely to experience asthma exacerbation requiring corticosteroids or hospitalization [11]. When the asthmatic patient groups were divided into smaller groups based on vitamin D levels, 72.2% were deficient while 27.8% had adequate levels; they found that uncontrolled asthma rates were higher in the group with insufficient vitamin D (p<0.01) [11]. Even though Turkeli et al. did not test whether adjusting the level of vitamin D through routine administration of vitamin D supplements improves the rate of exacerbation, they found a relationship between vitamin D levels and asthma exacerbation, thereby opening the conversation for further investigation [11].

In a two-phase cohort study by Solidoro et al., which included 119 adult patients with a confirmed diagnosis of asthma, they found low levels of vitamin D in most of the asthmatic patients (93%) ranging from 75.5% with less than 20ng/mL to 17.5% having less than 30ng/mL of 25-hydroxyvitamin D [12]. Puranik et al. reviewed three observational studies to investigate the relationship between asthma flare-ups and vitamin D levels in children and found that low levels of the vitamin were present in the children during asthma exacerbation (serum 25-hydroxyvitamin D < 30ng/ml)[1].

Furthermore, a review of evidence on the relationship between vitamin D and respiratory conditions such as asthma, chronic obstructive pulmonary disorder (COPD), and cystic fibrosis by Maes et al. found that, though vitamin D deficiency is prevalent in exacerbations of these conditions, vitamin D deficiency is less likely to be the cause of the exacerbations. The review also found that low vitamin D is associated with increased asthma exacerbations even though the exact mechanism of action for the influence remains unclear [7].

Han et al. conducted a cross-sectional study involving 678 eligible Puerto Rican children between the ages of six to 14 years to investigate the effect of a healthy diet on asthma exacerbation. They randomly selected the children from metropolitan households in San Juan (Puerto Rico) and assigned the children to one of six food groups of either fruit, vegetables, grains, proteins, dairy, or fats, and followed them from March 2009 to June 2010 [13]. Based on the analysis of the questionnaire completed by each child's parent, unhealthy diets and vitamin D deficiency were associated with more likelihood of having a severe asthma flare-up. Unlike children with healthy diets and adequate serum vitamin D levels, children having low serum levels of 25-hydroxyvitamin D [25(OH)D)] were also more likely to have asthma flare-ups, as well as hospital stays due to asthma flare-ups [odds ratio (OR) = 3.4, 95% confidence interval (CI) = 1.5 to 7.5] or ≥1 being hospitalized due to asthma (OR = 3.9, 95% CI = 1.6 to 9.8, OR = 3.4, 95% CI = 1.5 to 7.5) [13].

The studies considered here measured the vitamin D level in asthmatic patients during periods of asthma exacerbation and found that most of those patients had low levels of vitamin D. They were unable to establish whether asthma exacerbation led to the low levels of vitamin D or vitamin D deficiency caused the asthma flare-ups.

Effect of vitamin D supplementation on asthma exacerbation in children

Ducharme et al. performed a randomized clinical trial on 47 children aged one to five, by administering two oral doses of 100,000 IU vitamin D3 (treatment group) or identical placebo to the children three and half months apart, and they found no significant decrease in the rate of asthma flair-ups in the treatment group of 24 children as the doses of vitamin D raised the overall blood level of vitamin D metabolites over the seven months [14].

Furthermore, Luo et al., in a meta-analysis involving seven randomized controlled trials with a total of 903 asthmatic patients, investigated the effect of giving vitamin D along with asthma controllers and found no improvement in asthma exacerbation rates despite a significantly increased level of vitamin D in the participants [5]. The seven trials were done in different countries, namely Germany, Poland, Turkey, Hershey, India, the Netherlands, and the United Kingdom; three studies were conducted in children, while four studies were in adults, and the studies enrolled stable asthma, persistent asthma, and Immunoglobulin E (IgE)-dependent asthma, while different doses of vitamin D from multiple manufacturers were administered orally or subcutaneously [5].

Forno et al. performed a randomized controlled trial involving 192 participants, 77 girls, and 115 boys, with a mean age of 9.8 years, receiving 4000 UI vitamin D supplementation or placebo over 48 weeks along with fluticasone propionate maintenance and found that there was no significant difference between time to severe asthma exacerbation for the treatment group and the placebo group; there was no significant difference in the inhaled corticosteroid use during this time [15]. Wang et al. also found no significant change in exacerbation rate in children participants as seen in asthma control test scores, Fractional exhaled Nitric Oxide (FeNO), interleukin-10, and adverse events, following vitamin D supplementation, unlike in the adult population [16].

Also, Jensen et al., in a randomized, double-blinded, controlled trial with 22 asthmatic pre-school children of one to five years old, who were evenly divided into control group and intervention group and given daily doses of oral vitamin D (bolus 100,000 IU vitamin D3, and maintenance dose 400 IU vitamin D3 daily) over six months, found no significant difference in the rate of use of oral rescue inhalers between the intervention group or placebo group [group oral corticosteroids rates were 0.82 and 1.18/child, intervention versus control [relative risk (RR) = 0.68; 95 % CI = 0.30, 1.62; nonsignificant)] [17]. Although vitamin D levels improved for the participants at six months, they were unable to continue the study beyond that point due to lack of funding [17].

In contrast, Puranik et al. investigated the effect of supplementing vitamin D in asthmatic patients by considering two small, randomized studies that supplemented 500 to 2,000 IU/d of vitamin D to asthmatic patients and found a significant decrease in the rate of asthma exacerbation over six months [1].

Results from the above studies indicate that supplementing vitamin D in asthmatic children may not reduce the asthma exacerbation rates as there was strong evidence of a lack of responsiveness to vitamin D in asthmatic children. Only one of the eight studies considered here reported a reduction in asthma exacerbation; the remaining studies found no reduction in flare-ups with vitamin D supplementation in asthmatic children.

Effect of vitamin D supplementation on asthma exacerbation in adults

Jaura et al. performed a metanalysis on Cochrane systematic reviews of randomized controlled trials and found that supplementing vitamin D reduces asthma exacerbations [18]. The study observed that administering vitamin D to adult patients with mild-to-moderate asthma reduced the rate of exacerbations by 30% in vitamin D-deficient adults [25(OH)D < 25nmol/L; 92 participants in three randomized control trials (RCTs); number needed to treat (NNT) = 4.3]. However, they found no significant reduction in exacerbations for participants with higher baseline vitamin D levels (764 participants in six RCTs) [18]. Vitamin D supplementation led to reduced asthma exacerbation rate in patients having low baseline vitamin D levels (0.19 vs 0.42 events per participant-year; P = .046); an overall average of 900 IU/day of (range, 400-4000 IU/d) was administered to the participants in the randomized controlled trials [18]. This study showed that supplementing vitamin D is effective in reducing asthma exacerbations in vitamin D-deficient asthmatic adults.

Wang et al. performed a metanalysis with 14 randomized controlled trials and a total of 1421 participants (adults and children) receiving vitamin D and found a substantial reduction in the rate of asthma flare-up by 27% [relative risk (RR):0.73, 95% Cl = 0.58 to 0.92], but the significant reduction in asthma with vitamin D supplementation was mainly observed in adult participants (RR:0.75, 95% CI = 0.59, 0.95) [16]. This research delineates the effectiveness of supplementing vitamin D in asthmatic patients based on age, as younger patients did not experience improvements.

Additional support for the effectiveness of vitamin D supplementation in adult patients can be found in the second phase of the cohort study by Solidoro et al., which involves vitamin D supplementation (100, 000 IU IM x1 followed by 5,000 oral doses weekly, and 400 IU oral doses daily) over one year [12]. They found that for patients with initial vitamin D levels of less than 20ng/mL, there was a reduction in asthma exacerbation rate (from 2.6 ± 1.2 to 1.6 ± 1.1, p < 0.001), circulating eosinophils (from 395 ± 330 to 272 ± 212 106/L, p < 0.001), and need of oral corticosteroids courses (from 35 to 20, p = 0.007) and improvement of airway obstruction [12] unlike in asthmatic patients with higher initial levels of vitamin D. This research implies that supplementing vitamin D in asthmatic patients having low levels of vitamin D is beneficial for reducing flare-ups.

Jolliffe et al. conducted a systematic review involving a single-step meta-analysis with a total of 955 individual participants data (IPD) in seven studies and found that administering vitamin D supplements decreased the rate of asthma flare-ups in the participants (adjusted incidence rate ratio [aIRR]:0·74, 95% CI = 0·56 to 0·97; p = 0·03; 955 participants in seven studies; high-quality evidence); they also conducted a two-step IPD meta-analysis with 719 participants in four studies, and the asthma exacerbation rates were found to be reduced as well (aIRR:0·69, CI = 0·52 to 0·92, p = 0·01; p-heterogeneity = 0·56)[4]. However, the reductions in exacerbation were only significant in participants that had an initial vitamin D level of less than 25nmol/L (aIRR:0.33, 95% CI = 0.11 to 0.98; 92 participants in three studies; within subgroup p = 0.046), as participants with higher serum vitamin D level did not experience a signal reduction in asthma exacerbation rate (aIRR:0.77, CI = 0.58 to 1.03; 764 participants in six studies; within subgroup p=0.08) [4].

Martineau et al. conducted a systematic review of three RCTs involving 680 participants comprising 658 adults and 22 children and found that supplementing vitamin D reduces the rate of asthma flare-up requiring corticosteroid use in the participants (RR:0.63, 95% CI = 0.45 to 0.88; 680 participants; three studies; 14 high-quality evidence) while also reducing the tendency of having at least one flare-up requiring an emergency room visit or hospital stay or both (OR = 0.39, 95% CI = 0.19 to 0.78; NNT for an additional beneficial outcome = 27), but the severity of the flare-ups was not affected [19]. This research had predominantly adult populations.

A combined analysis of two randomized controlled trials by Ames et al. revealed that supplementing vitamin D ranging from 2400 IU/d to 4000 IU/d led to a reduction in the likelihood to have asthma flare-ups from zero to three years by 24% [adjusted odds ratio (aOR): 0.74, 95% CI = 0.57 to 0.96); participants with a basal level of greater than or equal to 30ng/ml 25(OH)D (aOR:0.54, 95% CI = 0.33 to 0.88) [20]. Ames et al. also found that supplementing 4000 IU/d vitamin D in a randomized controlled trial led to a similar increase in vitamin D levels for both African Americans and non-African Americans, while a metanalysis of individuals from seven randomized controlled trials with high-quality evidence also revealed a reduction in asthma exacerbation requiring systemic corticosteroids after taking vitamin D supplements by 26%. However, the lowering of asthma flare-up was more in individuals that had an initial vitamin D level of less than 10ng/mL (67% of the total 92 participants) [20].

In contrast, Camargo et al. conducted a post hoc analysis of data from a randomized, double-blinded, placebo-controlled trial, with monthly administration of vitamin D to older adults and found vitamin D supplementation to be ineffective in reducing the rate of asthma exacerbation in a sample of 775 patients that had either asthma or COPD at the start of the study in Auckland, New Zealand; the mean age of the participants was 67 years, 56% were male, and they were followed for an average period of 3.3 years while receiving 100,000 IU vitamin D monthly or placebo with an initial one-time dose of 200,000 IU vitamin D or placebo [21].

Also, van Brakel et al. performed a systematic review involving 30 articles describing 28 individual studies clustered based on six nutritional interventions - herbs, herbal mixtures and extracts, supplements, weight-loss, vitamin D, and Omega-3 LCPUFAs. They found no improvement in lung function in four out of five studies from the vitamin D3 supplementation cluster while none of the studies recorded an improvement in asthma control or quality of life with vitamin D supplements [2]. In the metanalysis involving seven randomized controlled trials by Luo et al. where four studies were in adults, the researchers found no improvement in asthma exacerbation following supplementation with vitamin D; the study findings were, however, based on a statistical analysis of the data from all the seven studies reviewed [5].

From the above-mentioned studies, supplementing vitamin D does not decrease asthma exacerbation rate in asthmatic adults except in those with low baseline levels of vitamin D. The characteristics of the studies reviewed are shown in Table 1.

**Table 1:** Study characteristics

Reference	Year	Study type	Sample size	Population	Follow-up time (mo)	Outcome(s)
Ames et al. [20]	2021	Metanalysis, randomized controlled trials	92	Adults	variable	Vitamin D supplements lead to significant reductions in the rate of asthma exacerbations in participants with low baseline levels of vitamin D
Camargo et al. [21]	2021	Randomized controlled trials	775	Adults	40	Vitamin D supplementation was ineffective in reducing the rate of asthma exacerbations in the participants
Ducharme et al. [14]	2019	Randomized controlled trial	47	Children	7	No significant decrease in asthma exacerbation rates competent in the treatment
Forno et al. [15]	2020	Randomized controlled trials	192	Adults and children	12	No change in exacerbations after supplementing vitamin D
Han et al. [13]	2017	Cross-sectional study	678	Children (six to 18 years)	15	Vitamin D deficiency was associated with more likelihood of having severe asthma flare-ups
Jaura et al. [18]	2020	Metanalysis	764	Adults	variable	Vitamin D supplementation led to a reduced asthma exacerbation rate in patients having low baseline vitamin D levels
Jensen et al. [17]	2016	Randomized controlled trials	22	Children	6	No significant difference in rescue inhaler or corticosteroid uses between the treatment group and control group
Jolliffe et al. [4]	2017	Metanalysis	955	Adults	variable	Supplementing vitamin D reduces the rate of asthma flare-up requiring corticosteroid use in participants with low baseline levels of vitamin D
Luo et al. [5]	2015	Metanalysis of randomized controlled trials	903	Adults and children	variable	No improvement in asthma exacerbation with vitamin D supplementation
Martineau et al. [19]	2016	Systematic review	680	Adults and children	variable	Supplementing vitamin D reduces the rate of asthma flare-up requiring corticosteroid use in the participants
Puranik et al. [1]	2017	Observational studies	148	children	6	Low levels of the vitamin were present in the children during an asthma exacerbation. Supplementing Vitamin D in asthmatic children did not lead to a reduction in asthma exacerbation rates.
Solidoro et al. [12]	2017	Two-phase cohort study	119	Adults	12	Low levels of vitamin D are found in most asthma patients. A significant reduction in asthma exacerbation was found in participants with baseline low levels of vitamin D.
Turkeli et al. [11]	2016	Cross-sectional study	230	children	3	Patients with low levels of vitamin D were likely to have an asthma exacerbation
van Brakel et al. [2]	2020	Systematic reviews	2501	Adults and children	variable	No improvement in asthma control or quality of life with vitamin D supplements
Wang et al. [18]	2019	Randomized controlled trials	1421	Adults and children	variable	A significant reduction was found in adult asthmatic patients. No significant reduction in the pediatric participants.

Limitations

Our literature review has few limitations relating to the study population and different follow-up periods. There were fewer pediatric patients than adult patients in the studies with the mixed population, which could skew the findings to reflect adult population findings; so, we classified findings for the mixed studies as that of the adult population.

## Conclusions

We studied the relationship between vitamin D and worsening asthma attacks and found that low vitamin D levels are present in asthmatic patients during periods of asthma exacerbation. However, we did not find consistent evidence that supplementing vitamin D reduces asthma exacerbations in a pediatric population. In the adult population, supplementing vitamin D may lead to a statistically significant reduction of asthma exacerbation rates in asthmatic patients with low levels of vitamin D. High-quality, large, randomized trials may be needed to study the effect of supplementing vitamin D in pediatric patients with low levels of the vitamin.
